# Neurobiology of Stress-Induced Nicotine Relapse

**DOI:** 10.3390/ijms25031482

**Published:** 2024-01-25

**Authors:** Xinyu Wang, Yun Chen, Jing Dong, Jing Ge, Xiaoliu Liu, Jianfeng Liu

**Affiliations:** Institute of Brain Science and Advanced Technology, Hubei Province Key Laboratory of Occupational Hazard Identification and Control, School of Medicine, Wuhan University of Science and Technology, Wuhan 430065, Chinachenyuncy@wust.edu.cn (Y.C.); 18186214945@163.com (J.D.);

**Keywords:** nicotine, stress, relapse, reinstatement, neurobiology

## Abstract

Tobacco smoking is the leading cause of preventable death and disease. Although there are some FAD-approved medicines for controlling smoking, the relapse rate remains very high. Among the factors that could induce nicotine relapse, stress might be the most important one. In the last decades, preclinical studies have generated many new findings that lead to a better understanding of stress-induced relapse of nicotine-seeking. Several molecules such as α3β4 nicotinic acetylcholine receptor, α2-adrenergic receptors, cannabinoid receptor 1, trace amine-associated receptor 1, and neuropeptide systems (corticotropin-releasing factor and its receptors, dynorphine and kappa opioid receptor) have been linked to stress-induced nicotine relapse. In this review, we discuss recent advances in the neurobiology, treatment targets, and potential therapeutics of stress-induced nicotine relapse. We also discuss some factors that may influence stress-induced nicotine relapse and that should be considered in future studies. In the final section, a perspective on some research directions is provided. Further investigation on the neurobiology of stress-induced nicotine relapse will shed light on the development of new medicines for controlling smoking and will help us understand the interactions between the stress and reward systems in the brain.

## 1. Introduction

The tobacco epidemic remains one of the biggest public health threats in the world [1]. According to the Centers for Disease Control and Prevention, tobacco smoking is the leading cause of preventable death and disease in the United States. The situation is estimated to be worse under some conditions that increase stress and anxiety, such as COVID-19 lockdowns. It has been reported that pandemic lockdowns may be associated with increased daily tobacco consumption and relapse, which may even be sustained after lockdowns [2]. Nicotine is widely accepted as the key component of tobacco that produces addictive effects. In this regard, nicotine replacement therapy has been used as a common approach to assist smoking cessation worldwide [3]. Other pharmacological treatments for controlling smoking include the FDA-approved varenicline tartrate and bupropion hydrochloride, which do not contain nicotine. Although these currently available treatments could effectively, to some extent, help quit tobacco use, the relapse rate remains high. A study showed that the relapse rate of smoking within six months after abstinence was estimated to be as high as 75% [4]. Therefore, relapse has been the most critical barrier to the long-term success of smoking cessation.

The relapse of nicotine use could be triggered by many factors, including symptoms of nicotine withdrawal, weight gain, and stress [5,6,7,8,9]. Among them, stress is particularly important due to the natural characters of stress: common in daily life, unpredictable, highly variable, and occasionally disastrous [7,8]. These features of stress influence the function of the stress and reward systems in the brain and make the stress-triggered relapse of nicotine use a very complex problem [10,11,12,13]. In the last decades, accumulative studies have generated new findings that lead to a better understanding of stress-induced relapse of nicotine-seeking. This review discusses recent advances in the neurobiology, treatment targets, and potential therapeutics of stress-induced nicotine relapse. In the final section, a perspective on some directions that could be investigated in future studies is provided.

## 2. Experimental Models of Stress-Induced Nicotine Relapse

In humans, the reappearance of smoking behavior after abstinence could be classified into three concepts: a relapse, which was defined as seven consecutive days of smoking at least one puff per day following a period of total abstinence; a lapse, which could be defined as an isolated smoking episode; a relapse crisis, which could be defined as any situation in which the temptation to smoke occurs [14]. However, these concepts have not been distinguished in the currently available experimental models of nicotine relapse. In this review, nicotine relapse is simply referred to as the reappearance of nicotine-taking or nicotine-seeking behaviors after abstinence. Although human and animal models of stress-induced nicotine relapse were reported in the literature, the former was not widely used [15,16]. Most of our understanding of stress-induced nicotine relapse is from investigations using animal models [16,17,18].

### 2.1. Nicotine-Induced CPP and Nicotine Self-Administration

**Figure 1:**
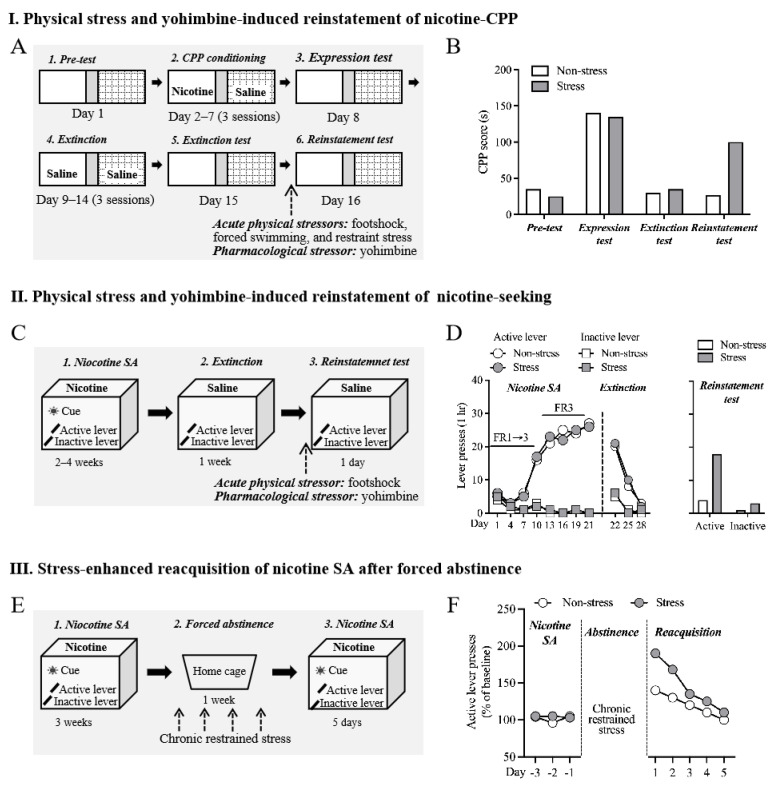
Animal models of stress-induced nicotine relapse. (**A**) Schematic procedure of physical stress- and yohimbine-induced reinstatement of nicotine CPP. Animals underwent pre-test, conditioning, expression test, extinction, extinction test, and reinstatement test of nicotine CPP. Acute physical stress or yohimbine were given immediately before the reinstatement test. (**B**) Representative of behavioral data. Stressed animals showed a higher level of preference for the nicotine-paired side during the reinstatement test. (**C**) Schematic procedure of physical stress- and yohimbine-induced reinstatement of nicotine-seeking. Animals underwent nicotine self-administration, extinction, and reinstatement test. Acute physical stress or yohimbine were given immediately before the reinstatement of the nicotine-seeking test. Active but not inactive lever was paired with intravenous nicotine administration. (**D**) Representative of behavioral data. Stressed animals showed an increase in active lever presses during the reinstatement test. (**E**) Schematic procedure of stress-enhanced reacquisition of nicotine SA after forced abstinence. Animals underwent long-access nicotine self-administration (23 h/day), forced abstinence in the home cage, and nicotine self-administration again (reacquisition). Chronic restrained stress was conducted during the force abstinence period. (**F**) Representative of behavioral data. Compared to non-stressed animals, stressed animals showed higher levels of active presses during the reacquisition phase. CPP: conditioned place preference; SA: self-administration.

Currently available animal models of stress-induced relapse are established based on drug-induced conditioned place preference (CPP) and drug self-administration, the two widely used behavioral tasks to assess the rewarding and reinforcing effects of drugs (Figure 1) [19,20,21]. Although these animal models have similar processes, i.e., conditioning/training, extinction, and stress-induced reinstatement, they are fundamentally distinct. Nicotine-induced CPP is a typical Pavlovian conditioning paradigm in that a contextual CS (conditioned stimulus) is associated with the US (unconditioned stimulus), i.e., the euphoria experience of drug use. Nicotine self-administration is an operant conditioning paradigm in which active operant behavior (response) is reinforced by drugs of abuse (outcome) [22,23].

Here, we briefly describe the typical processes of these models (Figure 1). (1) Stress-induced reinstatement of nicotine CPP: for the conditioning of nicotine-induced CPP, an animal is confined in one side of the CPP chamber after injection of the drug, whereas the animal receives an injection of saline or vehicle when confined in the other side of the CPP chamber. After several association trainings, the animal will exhibit a preference for the nicotine-associate context during the CPP test, which is revealed by the longer time spent in the nicotine-paired context than the non-paired side. Extinction will occur after repeated exposure to the context without drug pretreatment, and the animal does not show a preference for the nicotine-paired side anymore after successful extinction. The preference can be triggered after the animal receives physiological, psychological, or pharmacological stress, termed stress-induced reinstatement of nicotine CPP. (2) Stress-induced reinstatement of nicotine-seeking: an animal is trained to actively press a lever or nosepoke that is paired with nicotine to obtain an injection of nicotine in the nicotine self-administration model. Extinction is then induced by replacing saline with nicotine injection when the animal undergoes self-administration. The response to the active lever or nosepoke can be reinstated when the animal experiences stress. This behavioral phenomenon is termed stress-induced reinstatement of nicotine-seeking behavior.

### 2.2. Physiological Stress-Induced Nicotine Relapse

Different types of physical stressors, including footshock, forced swimming, and restraint stress, have been reported to induce nicotine relapse in preclinical models. The first type of stress used under the experimentation condition to trigger drug relapse was footshock, a physical stressor utilized in many other behavioral tasks such as fear conditioning, active and passive avoidance, learned helplessness, and so forth [24,25,26]. Several pioneering studies showed that, comparable with the effects of the drug itself, a brief exposure to intermittent footshocks could reinstate the responding to drug-paired lever in the drug self-administration model, providing the first conclusive evidence that physical stress could reinstate drug-seeking behavior in animals [16,17,18,27,28]. Notably, many studies have confirmed that intermittent footshock stimuli can reliably induce the reinstatement of nicotine-seeking in rodents [18,29,30].

Besides self-administration, nicotine-induced conditioned place preference (CPP) has been widely used in previous studies. However, to the best of our knowledge, stress-induced reinstatement of nicotine CPP was not reported until 2008 [31]. It was shown in the study that acute restrained stress (30 min duration, immediately before CPP test) applied either 1 or 15 days after the extinction of nicotine CPP could lead to a robust reinstatement in rats [31]. Consistently, restraint stress, when applied immediately before the CPP test, is also a potent factor that could induce reinstatement of nicotine CPP in mice [32]. Later, it was shown that intermittent footshocks could also induce the expression of extinguished nicotine CPP [33]. Together, these data indicated that stress could reliably reinstate the nicotine-seeking in the nicotine SA model and the nicotine CPP in rodents.

### 2.3. Yohimbine-Induced Reinstatement of Nicotine-Seeking and Nicotine CPP

A large body of evidence indicates the noradrenergic system that originated from the locus coeruleus is crucial for the stress response, stress-related disorders, and drug addiction [34,35,36]. In particular, several agents targeting the noradrenergic system have been reported to be able to reinstate drug-seeking behaviors [37]. The most well-studied pharmacological stressor that could induce the reinstatement of nicotine-seeking is the α2-adrenergic receptor antagonist yohimbine [37]. There is extensive evidence that yohimbine has anxiogenic properties [38,39,40]. When administered systematically, yohimbine can induce anxiety in humans and rodents. Studies have demonstrated that yohimbine could generally lead to the reinstatement of drug-seeking behavior associated with many types of drugs of abuse, including nicotine. It was shown that yohimbine could lead to the reinstatement of nicotine-seeking when injected alone and potentiate cue-induced reinstatement when administered with cue presentation [30,33,41,42,43]. Furthermore, yohimbine could also induce the expression of nicotine CPP after extinction [33]. Consistently, systemic administration of yohimbine could reinstate the extinguished nicotine-induced CPP [33]. In a double-blinded, placebo-controlled, randomized crossover design study, it was shown that a combination of yohimbine and hydrocortisone could significantly increase nicotine-seeking behavior [9].

### 2.4. Stress-Enhanced Reacquisition after Extinction after Abstinence

Some animal studies showed that stress could enhance the reacquisition of nicotine self-administration after abstinence [44,45]. The studies revealed that repeated restraint stress (four sessions in total) during the abstinence from nicotine self-administration enhanced the subsequent reacquisition. This effect was not observed in animals that received limited stress (two sessions in total), suggesting that chronic repeated stress is required for this phenomenon [44]. It was demonstrated that the basolateral amygdala (BLA), ventral hippocampus (vHIPP), BLA-nucleus accumbense (NAc), and vHIPP-BLA circuits were essential for the effects of stress on the reacquisition of nicotine self-administration [45]. In addition, microinjection of positive allosteric modulators of GABA_A_ receptors into the BLA attenuated the reacquisition promotion effects of stress [45]. Therefore, these data provide evidence that the BLA could be a crucial brain area that mediated the stress-enhanced reacquisition of nicotine self-administration.

However, we argue here that the model indeed evaluates nicotine intake after abstinence rather reacquisition of nicotine self-administration. Reacquisition (or relearning) refers to additional training similar to the original learning process [46,47,48,49,50]. Notably, in the “reacquisition” models, the reacquisition/relearning of drug self-administration typically follows an extinction or punishment process that induces the suppression of original behavioral responses [47,48,49,51,52,53]. In other words, the reacquisition model requires an extinction or punishment process to diminish the operant behavioral response before the subsequent reacquisition process. However, in the study by Yu and colleagues, nicotine self-administration was reevaluated only after a period of forced abstinence. Since the operant behavior remained before the second self-administration (or intake) phase, there was indeed no occurrence of “reacquisition” in these studies. Notwithstanding, we think this model provides an alternative behavioral strategy to study nicotine relapse since it is distinct from the reinstatement models in that animals do not have access to the drug during the behavioral tests.

## 3. Therapeutic Targets for Stress-Induced Nicotine Relapse

Accumulative studies using the behavioral models discussed above have implicated many molecules that play crucial roles in stress-induced nicotine relapse. Here, we discuss pharmacotherapeutic targets that we believe have the potential for developing effective treatments for stress-induced nicotine relapse, e.g., α3β4 nAChR, α2-adrenergic receptors, CB1 receptor, TAAR1, and neuropeptide systems (Table 1).

### 3.1. α3β4 nAChR

Nicotinic acetylcholine receptors (nAChRs) are the primary receptors that nicotine directly binds to in the brain [63,64,65]. nAChRs are pentameric ligand-gated ion channels that are composed of α-subunits (α2-10) and/or β-subunits (β2-4). The direct evidence that nAChRs are involved in the rewarding effects of nicotine is from studies using animal brain slices and behavioral testings [66]. It was demonstrated that nicotine could directly activate the nAChRs on the ventral tegmental area (VTA) neurons to modulate the firing of dopamine (DA) neurons and thereby influence the DA transmission in the NAc, one of the key projection brain areas of the VTA DA neurons [67,68]. The role of nAChR subtypes in the rewarding, reinforcing, and adverse effects of nicotine and nicotine addiction has been systemically introduced in many reviews (for example, please see reviews [69,70,71,72,73]).

Among these nAChRs, the α7 nAChR is the most common homomeric subtype and exerts a relatively low affinity for nicotine. In contrast, the α4β2 receptors are the most widely expressed heteromeric subtypes that are high-affinity for nicotine in the brain [73]. Indeed, different subunit-containing nAChRs might be distinctively involved in certain aspects of the positive and negative effects of nicotine. For example, there are several lines of evidence that the presynaptic α7 nAChR regulates the release of excitatory neurotransmitters in the VTA to regulate dopamine transmission of DA neurons [74]. In contrast, α5-containing nAChR in the habenular participates in the inhibitory motivational signal of nicotine to control nicotine intake [75]. An α4 mutant mice line showing hypersensitivity to nicotine showed enhancement in nicotine-induced CPP and was sensitive to nicotine-induced hypothermia and tolerance, suggesting that α4 subtype activation was sufficient for nicotine reward, tolerance, and sensitization [76]. Consistently, selective deletion of α4 in the DA neurons abolished the anxiolytic effects of nicotine and prevented nicotine-induced CPP [77]. The α3-containing nAChRs have been linked to the rewarding effects of nicotine. Using whole-cell recordings, a study showed that α3β4 nAChR antagonist α-conotoxin AulB could completely prevent nicotine-induced activation of medial habenula neurons [78]. Notably, the α3β4 nAChR partial agonist AT-1001 could dose-dependently attenuate yohimbine-induced reinstatement of nicotine-seeking after extinction [54]. In addition, AT-1001 did not induce robust withdrawal symptoms as the nicotine receptor antagonist mecamylamine did in the experiment, suggesting that AT-1001 might be a promising treatment for nicotine dependence [54]. The α3β4 nAChR is predominantly expressed in the medial habenula (MHb), interpeduncular nucleus (IPN), and the fasciculus retroflexus (fr) [79,80]. It was suggested that the α3β4 nAChRs in the MHb-IPN pathway may be critical in mediating nicotine reward and withdrawal [81]. However, the role of α3β4 and other nAChR in stress-induced nicotine relapse remains understudied so far.

### 3.2. α2-Adrenergic Receptor

As mentioned above, mechanistic investigation on the footshock-induced reinstatement of drug-seeking implicated the involvement of the noradrenaline (NE) system in the stress-induced reinstatement of drug-seeking. Almost all of the adrenergic receptor subtypes (α1-, α2-, β1-, β2-, β3-adrenergic receptors) are reported to be involved in reward and drug relapse. For example, it was reported that microinjection of the β1- and β2-receptor antagonists betaxolol and ICI-118,551 into the bed nucleus of the stria terminalis (BNST) or central amygdala (CeA) could block footshock- but not priming-induced reinstatement of cocaine-seeking [82]. The α1 antagonist prazosin at a certain dosage could dose-dependently reduce nicotine self-administration without affecting food consumption [83]. Prazosin could also reduce cue- and priming-induced reinstatement of nicotine-seeking [83]. Among all the adrenergic receptors, the α2-adrenergic receptor may be the best-studied adrenergic receptor in stress-induced nicotine relapse.

It was shown that the α2-adrenergic receptor agonists clonidine, lofexidine, and guanabenz could prevent footshock-induced NE release in the locus coeruleus LC-projecting brain regions PFC and amygdala [84]. Systemic administration of these compounds attenuated footshock- but not priming-induced reinstatement of cocaine-seeking, suggesting a selective role of the α2-adrenergic receptor in stress-induced drug relapse [84]. Consistently, systemic administration of clonidine could significantly reduce footshock-induced reinstatement of nicotine-seeking [43]. It should be mentioned that one concern for the inhibitory effect of clonidine is that the lowest effective dose of clonidine on nicotine relapse slightly but significantly decreased response for chocolate-flavored food pellets, suggesting a potential non-specific inhibition [43]. Further study showed that intra-CeA administration of the α2-adrenergic receptor agonists clonidine and dexmedetomidine, but not the nonselective β1/β2-adrenergic receptor antagonist propranolol or the α1-adrenergic receptor antagonist prazosin, attenuated footshock-induced reinstatement of nicotine-seeking [42,43]. Furthermore, the inhibitory effects of intra-CeA clonidine and dexmedetomidine were not due to non-specific motor inhibition since local administration of these compounds did not affect food self-administration [42]. Taken together, these data suggested that the α2-adrenergic receptor in the CeA might be important for stress-induced nicotine relapse.

### 3.3. CB1 Receptor

The endocannabinoid system has been greatly implicated in drug addiction [85,86,87]. The cannabinoid receptors include the CB1 receptor, CB2 receptor, and a putative CB3 receptor (GPR55). Although there is some evidence that pharmacologically modulating activities of the CB2 (the potential CB2 receptor agonist β-Caryophyllene) and GPR55 (the GPR55 agonist O-1602) could influence nicotine intake and nicotine-seeking [88,89], the CB1 receptor attracted the most interest from researchers in the field.

Many preclinical and clinical studies showed that CB1 receptor antagonists/inverse agonists could attenuate the rewarding and reinforcing effects of nicotine, nicotine intake, and relapse [90,91,92,93,94]. For example, the cannabinoid-1 receptor inverse agonist rimonabant could significantly attenuate nicotine-induced dopamine release, nicotine self-administration, and the cue-induced reinstatement of nicotine- and sucrose-seeking behaviors [90,95]. Rimonabant had been approved as an anti-obesity medicine but was withdrawn later due to its adverse psychiatric side effects [96,97,98]. Other CB1 ligand, such as the CB1 neutral antagonist AM4113, was also reported to be potential avenues for the treatment of nicotine use. AM4113 was shown to be a potential medicine for nicotine addiction regardless of addition state since it could effectively reduce a wide range of nicotine addiction-related behaviors, including nicotine-taking, motivation for nicotine, and cue- and drug-induced reinstatement of nicotine-seeking [55]. AM4113 could block nicotine-induced firing of dopaminergic neurons in the VTA [55]. Consistently, intra-VTA but not intra-NAc administration of the CB1 antagonist AM215 could block nicotine intake [99]. Further study confirmed the inhibitory effects of AM4113 on the maintenance of nicotine self-administration and cue-, drug-, and yohimbine-induced reinstatement of nicotine-seeking [55,100]. These data suggest that AM4113 could be a potent medicine to help quit nicotine use and stress-induced nicotine relapse.

### 3.4. Trace Amine-Associated Receptor 1 (TAAR1)

TAAR1 is the best-well-characterized receptor of trace amine, a group of animal amines expressed at the nalo molar level in the mammalian brain [101,102,103]. Since its first clone in 2001, TAAR1 has been reported to be involved in many brain functions and brain disorders, including drug addiction [104,105]. TAAR1 is mainly expressed in the monoaminergic system in the brain, including the VTA, dorsal raphe, dorsal and ventral striatum, and prefrontal cortex [101,102,103]. Electrophysiological and electrochemical data indicated that TAAR1 could negatively modulate monoaminergic transmission [106,107].

There is growing evidence that the selective TAAR1 agonists could be potential therapeutics for addictions to amphetamines, cocaine, nicotine, and opioids [101]. Studies have also indicated the anti-stress properties of TAAR1 agonists [106,108]. For example, TAAR1 agonists could attenuate stress-induced hyperthermia and stress-induced cognitive impairment [108,109]. To date, only three studies have evaluated the effects of TAAR1 agonists on nicotine-related behaviors and demonstrated the inhibitory effects of the TAAR1 agonists on nicotine addiction, relapse to nicotine, and nicotine withdrawal [110,111,112]. In particular, our unpublished data showed that the TAAR1 partial agonist RO5263397 could effectively reduce footshock-induced reinstatement of nicotine CPP (unpublished observation). However, the role of TAAR1 and the effects of its agonists on stress-induced nicotine relapse remain largely unknown. Further studies are needed to evaluate the effects of other TAAR1 agonists on nicotine-induced relapse induced by different types of stress and to investigate the underlying mechanisms.

### 3.5. Neuropeptide Systems

Accumulative evidence has suggested that the neuropeptide systems are crucial in the development of drug addiction and relapse [113,114]. For example, a human study showed that, during the withdrawal period from nicotine use, relapsers showed lower levels of orexin (also known as hypocretin) and ghrelin but a higher level of peptide YY in the blood [115,116]. Animal studies have suggested that different neuropeptides may specifically mediate certain aspects of the behavioral effects of nicotine. Neuropeptides such as corticotropin-releasing factor (CRF) ghrelin, neurotensin, and orexin could be important for the rewarding effects, whereas, CRF, neuropeptide Y, and oxytocin might be involved in nicotine withdrawal [117] (for more details, see a systemic review [118]). Despite the increased attention on the role of neuropeptides in addiction, only a few studies have investigated the role of neuropeptide systems in stress-induced nicotine relapse, and the findings on some neuropeptides are mixed. The most well-studied neuropeptide systems in mediating stress-induced nicotine relapse are CRF and dynorphin and their receptors.

### 3.6. CRF

CRF neurons have been found in various brain regions, predominantly in the paraventricular nucleus of the hypothalamus but also in other areas such as the cortex, hippocampus, VTA, and extended amygdala [119,120,121]. CRF exerts its function via two receptors: CRF1 and CRF2, which are expressed in the brain and peripheral, with particular expression patterns [122]. Extensive evidence has indicated that CRF is a key molecule that modulates stress response and the negative reinforcing effects of various drugs of abuse, including nicotine [123,124,125,126]. In particular, several studies have evaluated the effects of CRF receptor antagonists on the stress-induced reinstatement of nicotine-seeking.

Systemic administration of the CRF1 antagonist antalarmin blocked footshock stress-induced reinstatement of nicotine-seeking in mice [56]. Intra-cerebroventricular administration of the CRF1/2 receptor antagonist D-Phe CRF_(12–41)_ at the dosage that had no inhibitory effect on responding to chocolate-flavored food pellets could decrease footshock-induced reinstatement of nicotine-seeking in the subgroup of stress-responsive rats [43]. Moreover, intra-cerebroventricular injection of the CRF1 antagonist R278995/CRA0450, but not the CRF2 antagonist astressin 2B, dose-dependently reduced footshock-induced reinstatement of nicotine-seeking [127]. In the same study, R278995/CRA0450 but not astressin 2B decreased the nicotine receptor antagonist mecamylamine-potentiated brain reward thresholds in nicotine-dependent rats [127]. In sum, these studies might indicate that CRF1, but not CRF2, is a critical target mediating nicotine withdrawal-associated anhedonic state and stress-induced reinstatement of nicotine-seeking.

### 3.7. Dynorphin and Kappa Opioid Receptor (KOR)

Many studies showed that stress could cause the release of dynorphin, a peptide that activates the KOR within the monoaminergic systems to influence drug addiction [57,128]. Several lines of evidence support the involvement of the dynorphine-KOR system in nicotine addiction and nicotine relapse [129].

Firstly, KOR is important for the stress-enhance expression of nicotine-induced CPP. Similar to the effects of swimming stress, the kappa receptor agonist U50,488, when administered one hour before the test, dose-dependently potentiated nicotine CPP expression [130]. In contrast, pretreatment of KOR antagonist nor-BNI could block both the effects of swimming stress and KOR activation on CPP expression [130]. In addition, microinjection of nor-BNI into the BLA prevented U50488-enhanced nicotine CPP, suggesting that KOR in the BLA could be critical in mediating the effects [130]. Secondly, pharmacological activation of KOR is sufficient to trigger the reinstatement of nicotine-induced CPP [131]. Thirdly, pharmacological inhibition of KOR could prevent stress-induced reinstatement of nicotine relapse-like behaviors. A study demonstrated that systemic administration of the selective KOR antagonist norbinaltorphimine (nor-BNI) selectively blocked the reinstatement of nicotine CPP induced by forced swimming but not cue [58]. Consistent with this, another study showed that systemic administration of nor-BNI significantly attenuated footshock stress- and yohimbine- but not priming- induced reinstatement of nicotine CPP [33]. The study further showed that the KOR activity in the BLA was essential for yohimbine- and footshock stress-induced reinstatement of nicotine CPP [33]. In the nicotine self-administration model, it was shown that nor-BNI could selectively prevent yohimbine- but not cue-induced reinstatement of nicotine-seeking [59]. Lastly, genetic deletion of KOR or dynorphin could completely prevent the yohimbine-induced reinstatement of nicotine CPP [33].

### 3.8. Other Peptide Systems

Other neuropeptides, including orexin, oxytocin, and melanocortin 4 (MC4) receptors, have also been linked to nicotine addiction and/or stress-induced nicotine relapse. Orexin, a neuropeptide that has been linked to emotion, sleep, and stress response [132,133,134,135], may be a potential therapeutic target for controlling nicotine use [136,137]. Intra-cerebroventricular injection of orexin-1 could reinstate extinguished nicotine-seeking behavior, suggesting that orexin-1 might be involved in nicotine relapse [56]. However, the orexin-1 receptor blockade had no effects on stress-induced reinstatement of nicotine-seeking, suggesting that the orexin-1 receptor might not be involved in stress-induced nicotine relapse [56]. A recent systemic review that analyzed the effectiveness of oxytocin, a peptide that was proposed to be a potential therapeutic for addiction to various drugs of abuse, concluded that there is no firm conclusion that oxytocin could effectively treat drug addiction [138]. A recent clinical trial showed that intranasal administration of oxytocin did not change stress-induced nicotine craving assessed by subjective and physiological responses to stress in daily smokers [60]. Insulin has also been linked to nicotine relapse. A study showed that intranasal insulin reduced nicotine cravings and increased circulating cortisol levels [61]. There is evidence that the MC4 receptor might mediate the stress-induced nicotine relapse. A study showed that the MC4 receptor antagonists HS014 and HS024 could prevent stress-induced reinstatement of nicotine-seeking [30]. Interestingly, these MC4 antagonists did not affect the elevations in the intracranial self-stimulation thresholds precipitated by the nAChR antagonist mecamylamine, a procedure to evaluate the dysphoria associated with acute nicotine withdrawal [30]. These data thus suggested a selective role of the MC4 receptor in mediating stress-induced nicotine relapse but not in nicotine withdrawal-induced dysphoria.

Notably, the above-reviewed systems do not only regulate nicotine addiction but also play important roles in stress-associated physiological and psychological processes. It is known that the comorbidity of nicotine dependence and other psychiatric disorders is very high. For example, it is estimated that smokers are approximately twice as likely to have post-traumatic stress disorder (PTSD) than non-smokers in the general population, and individuals with PTSD are twice as likely to be current smokers than individuals without PTSD [139]. The comorbidity of nicotine dependence and other psychiatric disorders makes the health conditions more complicated and might influence the treatment outcomes. For example, although the α3β4 nAChR subtype partial agonist AT-1001 only produces weak withdrawal symptoms, this might be a major issue for subjects with comorbid psychiatry disorders because these patients might be more sensitive to innate stress response [54]. Thus, future studies should investigate whether the potential targets mentioned above are suitable for the prevention of stress-induced relapse when there is a comorbid disease condition.

## 4. Factors That Influence the Effects of Stress on Nicotine Relapse

Although the evidence reviewed above strongly supports that stress could generally trigger nicotine relapse, various factors that influence the behavioral and chemical responses to stress may affect the neural mechanism underlying the stress-induced nicotine relapse. Here, we discuss three factors that we believe are important: the properties of stress itself, individual response to stress, and sex-dependent response to stress.

Firstly, the effects of stress on nicotine relapse could be dramatically influenced by the properties of stress, e.g., the intensity, duration, stress type, and controllability of stress [131,140]. For example, the stressors used in the literature on stress-induced drug relapse were usually mild stress, which could generally increase behavioral responses to drug and reinstate drug relapse. However, studies showed that acute stress might contract with nicotine-induced chemical and electrophysiological alterations in the brain. It was shown that acute restraint stress could prevent nicotine-induced dopamine transmission in the NAc shell and nicotine-induced firing and bursting activity in the VTA [141,142]. It would be interesting to know whether this observation would be generalized to other types of stress. A study showed that single prolonged stress (SPS), a model used to mimic severely traumatic stress, reduced cocaine self-administration and cocaine CPP, indicating an anti-rewarding effect [140]. This effect of the SPS stressor is distinct from the promoting effects of the mild stressor footshock on cocaine self-administration [140]. Although the effects of SPS on nicotine reward and relapse are unclear, current data might suggest that traumatic stress might differently affect nicotine relapse compared to acute mild stressors. In addition, it was shown that chronic mild stress applied before the conditioning of nicotine-induced CPP blocked the reinstatement of nicotine CPP induced by the KOR agonist U50,488 [131]. Consistently, the KOR agonist U50,488 could cause reinstatement of nicotine CPP, which could be blocked by chronic mild stress [131]. Furthermore, subjects exposed to controllable stress may ameliorate stress-induced impairment of cognition and behaviors. Therefore, controllability might also be a factor that influences stress-induced nicotine relapse. Stress-induced behavioral and chemical responses could be affected largely by the properties of stress, which would ultimately influence its impact on nicotine relapse. However, further investigations are needed to uncover the detailed mechanisms.

Secondly, individual stress response or stress susceptibility may affect stress-induced nicotine relapse. In a study, rats were assigned into low- and high-stress response groups based on a cumulative score assessed by several stress-related tasks. Intriguingly, rats showed low- and high-stress responses responded differently to a single footshock-induced reinstatement of nicotine-seeking without showing any difference in nicotine self-administration acquisition or extinction [143]. The data thus suggest that the stress response trait of subjects could predetermine footshock-induced reinstatement of nicotine-seeking. Stress susceptibility could be influenced by genetic and epigenetic factors and stress experiences [144]. For example, early life stress could dramatically affect the stress response and coping strategies in later life [144]. Studies showed that early life stress is a common risk factor for various mental disorders, including addiction. Compared to individuals with low early life stress, human subjects with high levels of early life adversity showed greater levels of adrenocorticotropic hormone but lower levels of plasma and salivary cortisol levels in response to stress [145]. This indicated that early life adversity had a profound effect on stress-induced biological changes [145]. However, the impact of early life adverse stress on stress-induced nicotine relapse is understudied so far.

Thirdly, sex might be a crucial factor that influences stress-induced nicotine relapse [146]. Many studies have reported the sex-dependent effects of stress and stress hormones on tobacco use or the rewarding effects of nicotine. A clinical study demonstrated that lower saliva cortisol levels could predict relapse in men, whereas greater cortisol levels could predict relapse in women [147]. An animal study showed that nicotine could only dose-dependently induce CPP in male but not female animals, indicating sex-dependent conditioning of nicotine-induced CPP [148]. There is strong evidence that, compared to men, women are more likely to relapse after abstinence from smoking [149,150,151,152]. Particularly, women are less likely to quit tobacco use in response to stressful life events (i.e., health and financial events) compared to men [153]. An animal study showed that female rats, when compared to male rats, were more sensitive to the yohimbine-enhanced reinforcing efficacy of nicotine [154]. These data indicate that stress might be a principal factor that promotes tobacco use and relapse in females and that stress-induced relapse is strongly dependent on sex [5]. However, the sex-dependent effects of stress-induced relapse and its underlying mechanisms remain largely understudied, which should be investigated in future studies.

## 5. Social Stress-Induced Nicotine Relapse

Although this review focuses on findings from preclinical studies, we acknowledge that many human studies have provided important information on the neurobiology of stress-induced nicotine relapse. In particular, social stress has been recognized as a determinant factor that triggers smoking relapse and has received extensive attention for decades [155].

The interaction between social stress and smoking relapse is complex. It is widely accepted that a blunted response to social stress could predict relapse (please see Section 6.3 below). For example, a study showed that compared to non-smokers, smokers exhibited blunted stress response to acute stress that included public speaking, a kind of social stress, and math tasks [156]. As mentioned above, nicotine replacement therapy has effective treatment effects on preventing nicotine withdrawal and relapse; however, this treatment might induce some undesired responses to stress. Compared to the placebo patch, the treatment of the nicotine patch reduced social stress-induced hormonal response during withdrawal [157]. Because blunted stress response usually predicts a high rate of relapse, this study thus indicated a potential side effect of nicotine replacement [157]. It is worth noting that a clinical study showed that stress-related increases in risk-taking and attention failures, but not stress-related impulsivity, nicotine craving, or withdrawal, might predict early relapse to smoking in young adults. In a study, it was reported that psychosocial stress could elicit a high craving for nicotine, which could be reversed by some pharmacological treatment [61]. Taken together, these studies indicated that certain types of responses to social stress might be associated with smoking relapse [158]. However, this issue needs to be further investigated in future studies with larger cohorts of smokers.

## 6. Perspectives on Future Research Directions

Besides the urgent need to uncover the neurobiological underpinnings of stress-induced nicotine relapse, we propose here some other research directions on the effects of stress on nicotine relapse that should attract more attention.

### 6.1. The Brain System Actively Involved in Early Withdrawal

An interesting phenomenon is that although the relapse rate of smoking is very high within six months after abstinence, 60% to 70% of smokers who did not relapse within six months achieved successful cessation for at least eight years. A recent study in Korea, which included 463 participants, showed that among all participants who relapsed (72.8%), 82.2% of all relapses occurred within six months [159]. This clinical observation may imply that prevention of relapse within six weeks is critical for long-term smoking cessation. The time-dependent pattern should attract more attention in clinical treatments and mechanistic investigations in preclinical studies. We hypothesized that subjects with successful smoking cessation might develop particular mechanisms by recruiting a “coping system” in the brain for coping with the distress feelings associated with psychological withdrawal symptoms in the first six months. A potential interest could be identifying theses activated neural circuits or chemical changes that gradually developed during early abstinence. For example, there is a clinical study showing that the stress hormone ACTH in blood was increased across the abstinence period (4 weeks after abstinence) for successful quitters compared to relapsers [116]. Theoretically, promoting the “coping system” in the brain via pharmacological or psychological approaches would promote long-term abstinence and thus decrease the relapse rate. For example, a recent study showed that emotional intelligence training could help actively apply psychological coping strategies to combat smoking [160].

### 6.2. Stress-Induced Nicotine Relapse and Acute Withdrawal Symptoms

A large body of studies demonstrated that acute negative affective emotions associated with nicotine withdrawal contributed to the development of nicotine addiction and relapse [161,162,163]. Although a detailed review of the withdrawal on relapse is out of the scope of this review, we propose that acute withdrawal might interact with stress-induced nicotine relapse.

A human study designed to examine bupropion on stress response during nicotine withdrawal found that stress increased craving scores and withdrawal symptoms before abstinence but only had little effect on levels of these during abstinence, which might be due to high baseline levels of craving and withdrawal symptoms [62]. Bupropion reduced baseline levels of craving and withdrawal symptoms and decreased craving but not withdrawal symptoms scores under stress [62]. This study thus suggested that stress might have had greater effects on craving and withdrawal symptoms during ab libitum smoking but not abstinence. Although this study suggested that stress did not aggravate physical withdrawal symptoms after abstinence, we propose that acute withdrawal-induced anxiety might interact with stress to influence relapse potential. For example, a study showed that brain regions that could be predictive of relapse showed elevated responses to stress during nicotine [164].

Smokers who were deprived of cigarettes for 24 h showed higher startle responses during unpredictable but not predictable shocks compared to non-deprived subjects [165]. The study also revealed that self-reported negative withdrawal symptoms (e.g., anxiety, worry, irritability) were positively associated with a startle response. Because a predictable threat usually causes a fear response, whereas an unpredictable threat leads to an anxiety state, the study thus might indicate that nicotine withdrawal might selectively potentiate stress-associated anxiety but not fear [165]. In a study, it was shown that social stress-induced cortisol response in subjects who underwent nicotine withdrawal was stronger than normal smokers or nicotine replacement 12 h abstention with nicotine patch [157]. The result indicated either a greater hormonal stress response during nicotine withdrawal or dysregulation of the stress system after chronic nicotine treatment [157]. Another study showed that brain regions that might predict relapse displayed heightened neural responses to psychosocial stress during nicotine withdrawal, suggesting that nicotine withdrawal could alter neuron responses to psychosocial stress [164]. Nevertheless, the interaction between nicotine withdrawal-associated negative emotions and stress, especially social stressors, remains understudied.

### 6.3. Stress Response May Predict Relapse

Not only can stress influence nicotine use and relapse, but chronic smoking can also, in turn, alter stress response. For example, it was demonstrated that smokers exhibited blunted stress responses, revealed by a decrease in stress-induced salivary cortisol concentration and cardiovascular activity [156]. Growing evidence from studies on smokers suggests that a blunted stress response, indicated by attenuation in stress-induced chemical alterations in blood, could predict nicotine relapse. A human study that assessed the relationship between stress response during the early abstinence period and future relapse indicated that attenuation in psychological stress-induced increase in the adrenocorticotropin level was associated with early relapse [166]. Consistently, individuals who relapsed within four weeks showed attenuated hormonal (levels of ACTH and plasma cortisol) and cardiovascular (systolic and diastolic blood pressure) responses, indicating a blunted stress response in the relapsers [167]. Furthermore, a similar study found that although baseline levels of beta-endorphin were similar between relapsers and successful abstainers, stress-induced beta-endorphin response was attenuated in the relapsers at the beginning of their abstinence period [168]. These clinical studies thus provided solid evidence that a blunted stress response predicts early relapse, whereas a robust stress response predicts resiliency.

Stress-induced behavioral or psychological responses could be associated with nicotine relapse, which might be used as predictive phenotypes of nicotine relapse. For example, it is shown that stress-related increases in risk-taking and attention failures, but not impulsivity, nicotine craving, or withdrawal, could be associated with and thus might predict earlier relapse to smoking in young adult smokers [158]. Early prediction of nicotine relapse would help clinicians determine appropriate interventions for those potential relapsers. It should be noted that the meaning of these findings is limited due to the small population reported in these studies. The predictive effects of blunted stress response should be further evaluated in a large cohort of nicotine users. Another question for future research is whether interventions targeting blunted stress response could lead to successful smoking cessation.

## 7. Conclusions

Investigations using animal models, such as stress-induced reinstatement of nicotine-induced CPP and nicotine self-administration, have uncovered important neural mechanisms underlying stress-induced nicotine relapse. However, our understanding of this behavior remains inadequate. Clinical and preclinical studies indicate that stress is a high-risk or might be the primary factor that induces nicotine relapse. Therefore, there is an urgent need to further investigate the interaction between the stress and reward systems and the underlying neural mechanisms of stress-induced relapse.

## Figures and Tables

**Table 1:** Summary of pharmacological interventions on stress-induced nicotine relapse.

Target	Intervention	Stress Type	Nicotine Relapse-like Behavior	Effect	Reference
α3β4 nAChR	Partial agonist AT-1001 (s.c.)	Yohimbine	Reinstatement of nicotine-seeking	Reduced	[54]
α2-AR	Agonist clonidine (s.c.)	Footshock	Reinstatement of nicotine-seeking	Reduced	[43]
	Agonists clonidine and dexmedetomidine (intra-CeA)			Reduced	[42]
β1/β2-AR	Antagonist propranolol (intra-CeA)	Footshock	Reinstatement of nicotine-seeking	No effect	[42]
α1-AR	Antagonist prazosin (intra-CeA)	Footshock	Reinstatement of nicotine-seeking	No effect	[42]
CB1R	Neutral antagonist AM4113 (i.p.)	Yohimbine	Reinstatement of nicotine-seeking	Reduced	[55]
CRFR	CRF1/2R antagonist D-Phe CRF_(12–41)_ (i.c.v.)	Footshock	Reinstatement of nicotine-seeking	Reduced	[43]
	CRF1R antagonist antalarmin (s.c.)			Reduced	[56]
	CRF1R antagonist R2789/CRA0450 (i.c.v.)			Reduced	[57]
	CRF2R antagonist Astressin 2B (i.c.v.)			No effect	[57]
KOR	Antagonist nor-BNI (s.c.)	Forced swimming	Reinstatement of nicotine CPP	Reduced	[58]
	Nor-BNI (i.p.)	Footshock and yohimbine		Reduced	[33]
	Nor-BNI (intra-BLA)	Yohimbine		Reduced	[33]
	Nor-BNI (i.p.)	Yohimbine	Reinstatement of nicotine-seeking	Reduced	[59]
	KOR agonist U50,488 (i.p.)	NA		Induced	[59]
OX1R	Antagonist SB334867 (i.p.)	Footshock	Reinstatement of nicotine-seeking	No effect	[56]
	Orexin A peptide (i.c.v.)	NA		Induced	[56]
MC4R	Antagonists HS014 and HS024 (i.c.v.)	Footshock	Reinstatement of nicotine-seeking	Reduced	[30]
NA	Oxytocin (intranasal)	Psychosocial stress	Nicotine craving (human)	No effect	[60]
NA	Insulin (Intranasal)	Psychosocial stress	Nicotine cravings (human)	Reduced	[61]
NA	Bupropion	Psychosocial and physical stress	Nicotine cravings (human)	Reduced	[62]

Reinstatement of nicotine-seeking was assessed in the nicotine self-administration model in this table. Abbreviations: AR: adrenergic receptor; CB1R: cannabinoid 1 receptor; TAAR1: trace amine-associated receptor 1; CRFR: corticotropin-releasing factor receptor; KOR: kappa opioid receptor; MC4R: melanocortin-4 receptor; NA: not appreciated; nAChR: nicotinic acetylcholine receptor; nor-BNI: norbinaltorphimine; OX1R: orexin-1 receptor. Except for those clearly stated, findings on the nicotine-relapse-like behaviors were from animal studies in the table.

## Data Availability

Not applicable.

## References

[1] Wang T.W., Asman K., Gentzke A.S., Cullen K.A., Holder-Hayes E., Reyes-Guzman C., Jamal A., Neff L., King B.A. (2018). Tobacco Product Use Among Adults—United States, 2017. MMWR Morb. Mortal. Wkly. Rep..

[2] Gendall P., Hoek J., Stanley J., Jenkins M., Every-Palmer S. (2021). Changes in Tobacco Use During the 2020 COVID-19 Lockdown in New Zealand. Nicotine Tob. Res..

[3] Shahab L., Dobbie F., Hiscock R., McNeill A., Bauld L. (2017). Prevalence and Impact of Long-term Use of Nicotine Replacement Therapy in UK Stop-Smoking Services: Findings From the ELONS Study. Nicotine Tob. Res..

[4] Gorniak B., Yong H.H., Borland R., Cummings K.M., Thrasher J.F., McNeill A., Hyland A., Fong G.T. (2022). Do post-quitting experiences predict smoking relapse among former smokers in Australia and the United Kingdom? Findings from the International Tobacco Control Surveys. Drug Alcohol Rev..

[5] Torres O.V., O’Dell L.E. (2016). Stress is a principal factor that promotes tobacco use in females. Prog. Neuropsychopharmacol. Biol. Psychiatry.

[6] Bruijnzeel A.W. (2012). Tobacco addiction and the dysregulation of brain stress systems. Neurosci. Biobehav. Rev..

[7] Richards J.M., Stipelman B.A., Bornovalova M.A., Daughters S.B., Sinha R., Lejuez C.W. (2011). Biological mechanisms underlying the relationship between stress and smoking: State of the science and directions for future work. Biol. Psychol..

[8] Pomerleau O.F., Pomerleau C.S. (1991). Research on stress and smoking: Progress and problems. Br. J. Addict..

[9] Woodcock E.A., Stanley J.A., Diwadkar V.A., Khatib D., Greenwald M.K. (2020). A neurobiological correlate of stress-induced nicotine-seeking behavior among cigarette smokers. Addict. Biol..

[10] Lee J.H., Kang S., Maier S.U., Lee S.A., Goldfarb E.V., Ahn W.Y. (2023). Acute stress enhances memory and preference for smoking-related associations in smokers. Nicotine Tob. Res..

[11] Koob G.F. (2009). Dynamics of neuronal circuits in addiction: Reward, antireward, and emotional memory. Pharmacopsychiatry.

[12] Koob G.F., Vendruscolo L. (2023). Theoretical Frameworks and Mechanistic Aspects of Alcohol Addiction: Alcohol Addiction as a Reward Deficit/Stress Surfeit Disorder; Current Topics in Behavioral Neurosciences.

[13] Sharp B.M. (2019). Basolateral amygdala, nicotinic cholinergic receptors, and nicotine: Pharmacological effects and addiction in animal models and humans. Eur. J. Neurosci..

[14] Ossip-Klein D.J., Bigelow G., Parker S.R., Curry S., Hall S., Kirkland S. (1986). Classification and assessment of smoking behavior. Health Psychol..

[15] Oberleitner L.M.S., Moore K.E., Verplaetse T., Roberts W., McKee S.A. (2018). Developing a laboratory model of smoking lapse targeting stress and brief nicotine deprivation. Exp. Clin. Psychopharmacol..

[16] Erb S., Shaham Y., Stewart J. (1996). Stress reinstates cocaine-seeking behavior after prolonged extinction and a drug-free period. Psychopharmacology.

[17] Shaham Y., Stewart J. (1995). Stress reinstates heroin-seeking in drug-free animals: An effect mimicking heroin, not withdrawal. Psychopharmacology.

[18] Buczek Y., Le A.D., Wang A., Stewart J., Shaham Y. (1999). Stress reinstates nicotine seeking but not sucrose solution seeking in rats. Psychopharmacology.

[19] Jackson A., Bagdas D., Muldoon P.P., Lichtman A.H., Carroll F.I., Greenwald M., Miles M.F., Damaj M.I. (2017). In vivo interactions between alpha7 nicotinic acetylcholine receptor and nuclear peroxisome proliferator-activated receptor-alpha: Implication for nicotine dependence. Neuropharmacology.

[20] Mantsch J.R., Baker D.A., Funk D., Le A.D., Shaham Y. (2016). Stress-Induced Reinstatement of Drug Seeking: 20 Years of Progress. Neuropsychopharmacology.

[21] Le A.D., Li Z., Funk D., Shram M., Li T.K., Shaham Y. (2006). Increased vulnerability to nicotine self-administration and relapse in alcohol-naive offspring of rats selectively bred for high alcohol intake. J. Neurosci..

[22] Fredriksson I., Venniro M., Reiner D.J., Chow J.J., Bossert J.M., Shaham Y. (2021). Animal Models of Drug Relapse and Craving after Voluntary Abstinence: A Review. Pharmacol. Rev..

[23] Golden S.A., Jin M., Shaham Y. (2019). Animal Models of (or for) Aggression Reward, Addiction, and Relapse: Behavior and Circuits. J. Neurosci..

[24] Liu J., Totty M.S., Melissari L., Bayer H., Maren S. (2022). Convergent Coding of Recent and Remote Fear Memory in the Basolateral Amygdala. Biol. Psychiatry.

[25] Li C., Sun T., Zhang Y., Gao Y., Sun Z., Li W., Cheng H., Gu Y., Abumaria N. (2023). A neural circuit for regulating a behavioral switch in response to prolonged uncontrollability in mice. Neuron.

[26] Chudoba R., Dabrowska J. (2023). Distinct populations of corticotropin-releasing factor (CRF) neurons mediate divergent yet complementary defensive behaviors in response to a threat. Neuropharmacology.

[27] Stretch R., Gerber G.J. (1973). Drug-induced reinstatement of amphetamine self-administration behaviour in monkeys. Can. J. Psychol..

[28] Shaham Y., Rajabi H., Stewart J. (1996). Relapse to heroin-seeking in rats under opioid maintenance: The effects of stress, heroin priming, and withdrawal. J. Neurosci..

[29] Flores A., Maldonado R., Berrendero F. (2020). THC exposure during adolescence does not modify nicotine reinforcing effects and relapse in adult male mice. Psychopharmacology.

[30] Qi X., Yamada H., Corrie L.W., Ji Y., Bauzo R.M., Alexander J.C., Bruijnzeel A.W. (2015). A critical role for the melanocortin 4 receptor in stress-induced relapse to nicotine seeking in rats. Addict. Biol..

[31] Leao R.M., Cruz F.C., Planeta C.S. (2009). Exposure to acute restraint stress reinstates nicotine-induced place preference in rats. Behav. Pharmacol..

[32] Titomanlio F., Perfumi M., Mattioli L. (2014). Rhodiola rosea L. extract and its active compound salidroside antagonized both induction and reinstatement of nicotine place preference in mice. Psychopharmacology.

[33] Nygard S.K., Hourguettes N.J., Sobczak G.G., Carlezon W.A., Bruchas M.R. (2016). Stress-Induced Reinstatement of Nicotine Preference Requires Dynorphin/Kappa Opioid Activity in the Basolateral Amygdala. J. Neurosci..

[34] Kobayashi K., Shikano K., Kuroiwa M., Horikawa M., Ito W., Nishi A., Segi-Nishida E., Suzuki H. (2022). Noradrenaline activation of hippocampal dopamine D(1) receptors promotes antidepressant effects. Proc. Natl. Acad. Sci. USA.

[35] Bierwirth P., Stockhorst U. (2022). Role of noradrenergic arousal for fear extinction processes in rodents and humans. Neurobiol. Learn. Mem..

[36] Varodayan F.P., Patel R.R., Matzeu A., Wolfe S.A., Curley D.E., Khom S., Gandhi P.J., Rodriguez L., Bajo M., D’Ambrosio S. (2022). The Amygdala Noradrenergic System Is Compromised With Alcohol Use Disorder. Biol. Psychiatry.

[37] Shepard J.D., Bossert J.M., Liu S.Y., Shaham Y. (2004). The anxiogenic drug yohimbine reinstates methamphetamine seeking in a rat model of drug relapse. Biol. Psychiatry.

[38] Morgan C.A., Southwick S.M., Grillon C., Davis M., Krystal J.H., Charney D.S. (1993). Yohimbine-facilitated acoustic startle reflex in humans. Psychopharmacology.

[39] Browne R.G. (1981). Anxiolytics antagonize yohimbine’s discriminative stimulus properties. Psychopharmacology.

[40] Pellow S., Chopin P., File S.E. (1985). Are the anxiogenic effects of yohimbine mediated by its action at benzodiazepine receptors?. Neurosci. Lett..

[41] Feltenstein M.W., Ghee S.M., See R.E. (2012). Nicotine self-administration and reinstatement of nicotine-seeking in male and female rats. Drug Alcohol Depend..

[42] Yamada H., Bruijnzeel A.W. (2011). Stimulation of alpha2-adrenergic receptors in the central nucleus of the amygdala attenuates stress-induced reinstatement of nicotine seeking in rats. Neuropharmacology.

[43] Zislis G., Desai T.V., Prado M., Shah H.P., Bruijnzeel A.W. (2007). Effects of the CRF receptor antagonist D-Phe CRF(12–41) and the alpha2-adrenergic receptor agonist clonidine on stress-induced reinstatement of nicotine-seeking behavior in rats. Neuropharmacology.

[44] Yu G., Chen H., Sharp B.M. (2014). Amplified reacquisition of nicotine self-administration in rats by repeated stress during abstinence. Psychopharmacology.

[45] Yu G., Sharp B.M. (2015). Basolateral amygdala and ventral hippocampus in stress-induced amplification of nicotine self-administration during reacquisition in rat. Psychopharmacology.

[46] Biala G., Pekala K., Boguszewska-Czubara A., Michalak A., Kruk-Slomka M., Grot K., Budzynska B. (2018). Behavioral and Biochemical Impact of Chronic Unpredictable Mild Stress on the Acquisition of Nicotine Conditioned Place Preference in Rats. Mol. Neurobiol..

[47] Nic Dhonnchadha B.A., Szalay J.J., Achat-Mendes C., Platt D.M., Otto M.W., Spealman R.D., Kantak K.M. (2010). D-cycloserine deters reacquisition of cocaine self-administration by augmenting extinction learning. Neuropsychopharmacology.

[48] Achat-Mendes C., Nic Dhonnchadha B.A., Platt D.M., Kantak K.M., Spealman R.D. (2012). Glycine transporter-1 inhibition preceding extinction training inhibits reacquisition of cocaine seeking. Neuropsychopharmacology.

[49] Sticht M., Mitsubata J., Tucci M., Leri F. (2010). Reacquisition of heroin and cocaine place preference involves a memory consolidation process sensitive to systemic and intra-ventral tegmental area naloxone. Neurobiol. Learn. Mem..

[50] Lu L., Xu N.J., Ge X., Yue W., Su W.J., Pei G., Ma L. (2002). Reactivation of morphine conditioned place preference by drug priming: Role of environmental cues and sensitization. Psychopharmacology.

[51] Su R.B., Wang W.P., Lu X.Q., Wu N., Liu Z.M., Li J. (2009). Agmatine blocks acquisition and re-acquisition of intravenous morphine self-administration in rats. Pharmacol. Biochem. Behav..

[52] Evans S.M., Foltin R.W., Hicks M.J., Rosenberg J.B., De B.P., Janda K.D., Kaminsky S.M., Crystal R.G. (2016). Efficacy of an adenovirus-based anti-cocaine vaccine to reduce cocaine self-administration and reacqusition using a choice procedure in rhesus macaques. Pharmacol. Biochem. Behav..

[53] Broomer M.C., Bouton M.E. (2023). A comparison of renewal, spontaneous recovery, and reacquisition after punishment and extinction. Learn. Behav..

[54] Yuan M., Malagon A.M., Yasuda D., Belluzzi J.D., Leslie F.M., Zaveri N.T. (2017). The alpha3beta4 nAChR partial agonist AT-1001 attenuates stress-induced reinstatement of nicotine seeking in a rat model of relapse and induces minimal withdrawal in dependent rats. Behav. Brain Res..

[55] Gueye A.B., Pryslawsky Y., Trigo J.M., Poulia N., Delis F., Antoniou K., Loureiro M., Laviolette S.R., Vemuri K., Makriyannis A. (2016). The CB1 Neutral Antagonist AM4113 Retains the Therapeutic Efficacy of the Inverse Agonist Rimonabant for Nicotine Dependence and Weight Loss with Better Psychiatric Tolerability. Int. J. Neuropsychopharmacol..

[56] Plaza-Zabala A., Martin-Garcia E., de Lecea L., Maldonado R., Berrendero F. (2010). Hypocretins regulate the anxiogenic-like effects of nicotine and induce reinstatement of nicotine-seeking behavior. J. Neurosci..

[57] Bruijnzeel A.W. (2009). kappa-Opioid receptor signaling and brain reward function. Brain Res. Rev..

[58] Jackson K.J., McLaughlin J.P., Carroll F.I., Damaj M.I. (2013). Effects of the kappa opioid receptor antagonist, norbinaltorphimine, on stress and drug-induced reinstatement of nicotine-conditioned place preference in mice. Psychopharmacology.

[59] Grella S.L., Funk D., Coen K., Li Z., Le A.D. (2014). Role of the kappa-opioid receptor system in stress-induced reinstatement of nicotine seeking in rats. Behav. Brain Res..

[60] Hamidovic A., Khafaja M., Brandon V., Anderson J., Ray G., Allan A.M., Burge M.R. (2017). Reduction of smoking urges with intranasal insulin: A randomized, crossover, placebo-controlled clinical trial. Mol. Psychiatry.

[61] Kearns N.T., Carl E., Stein A.T., Vujanovic A.A., Zvolensky M.J., Smits J.A.J., Powers M.B. (2018). Posttraumatic stress disorder and cigarette smoking: A systematic review. Depress. Anxiety.

[62] Kotlyar M., Drone D., Thuras P., Hatsukami D.K., Brauer L., Adson D.E., al’Absi M. (2011). Effect of stress and bupropion on craving, withdrawal symptoms, and mood in smokers. Nicotine Tob. Res..

[63] Sansone L., Milani F., Fabrizi R., Belli M., Cristina M., Zaga V., de Iure A., Cicconi L., Bonassi S., Russo P. (2023). Nicotine: From Discovery to Biological Effects. Int. J. Mol. Sci..

[64] Kim K., Picciotto M.R. (2023). Nicotine addiction: More than just dopamine. Curr. Opin. Neurobiol..

[65] Chen Z., Liu X.A., Kenny P.J. (2023). Central and peripheral actions of nicotine that influence blood glucose homeostasis and the development of diabetes. Pharmacol. Res..

[66] Picciotto M.R., Kenny P.J. (2021). Mechanisms of Nicotine Addiction. Cold Spring Harb. Perspect. Med..

[67] Calabresi P., Lacey M.G., North R.A. (1989). Nicotinic excitation of rat ventral tegmental neurones in vitro studied by intracellular recording. Br. J. Pharmacol..

[68] Pidoplichko V.I., DeBiasi M., Williams J.T., Dani J.A. (1997). Nicotine activates and desensitizes midbrain dopamine neurons. Nature.

[69] Le Houezec J. (1998). Nicotine: Abused substance and therapeutic agent. J. Psychiatry Neurosci..

[70] Dani J.A., Ji D., Zhou F.M. (2001). Synaptic plasticity and nicotine addiction. Neuron.

[71] Laviolette S.R., van der Kooy D. (2004). The neurobiology of nicotine addiction: Bridging the gap from molecules to behaviour. Nat. Rev. Neurosci..

[72] Dani J.A., De Biasi M. (2001). Cellular mechanisms of nicotine addiction. Pharmacol. Biochem. Behav..

[73] Gotti C., Zoli M., Clementi F. (2006). Brain nicotinic acetylcholine receptors: Native subtypes and their relevance. Trends Pharmacol. Sci..

[74] Nomikos G.G., Schilstrom B., Hildebrand B.E., Panagis G., Grenhoff J., Svensson T.H. (2000). Role of alpha7 nicotinic receptors in nicotine dependence and implications for psychiatric illness. Behav. Brain Res..

[75] Fowler C.D., Lu Q., Johnson P.M., Marks M.J., Kenny P.J. (2011). Habenular alpha5 nicotinic receptor subunit signalling controls nicotine intake. Nature.

[76] Tapper A.R., McKinney S.L., Nashmi R., Schwarz J., Deshpande P., Labarca C., Whiteaker P., Marks M.J., Collins A.C., Lester H.A. (2004). Nicotine activation of alpha4* receptors: Sufficient for reward, tolerance, and sensitization. Science.

[77] McGranahan T.M., Patzlaff N.E., Grady S.R., Heinemann S.F., Booker T.K. (2011). alpha4beta2 nicotinic acetylcholine receptors on dopaminergic neurons mediate nicotine reward and anxiety relief. J. Neurosci..

[78] Elayouby K.S., Ishikawa M., Dukes A.J., Smith A.C.W., Lu Q., Fowler C.D., Kenny P.J. (2021). alpha3* Nicotinic Acetylcholine Receptors in the Habenula-Interpeduncular Nucleus Circuit Regulate Nicotine Intake. J. Neurosci..

[79] Quick M.W., Ceballos R.M., Kasten M., McIntosh J.M., Lester R.A. (1999). Alpha3beta4 subunit-containing nicotinic receptors dominate function in rat medial habenula neurons. Neuropharmacology.

[80] Perry D.C., Xiao Y., Nguyen H.N., Musachio J.L., Davila-Garcia M.I., Kellar K.J. (2002). Measuring nicotinic receptors with characteristics of alpha4beta2, alpha3beta2 and alpha3beta4 subtypes in rat tissues by autoradiography. J. Neurochem..

[81] Leslie F.M., Mojica C.Y., Reynaga D.D. (2013). Nicotinic receptors in addiction pathways. Mol. Pharmacol..

[82] Leri F., Flores J., Rodaros D., Stewart J. (2002). Blockade of stress-induced but not cocaine-induced reinstatement by infusion of noradrenergic antagonists into the bed nucleus of the stria terminalis or the central nucleus of the amygdala. J. Neurosci..

[83] Forget B., Wertheim C., Mascia P., Pushparaj A., Goldberg S.R., Le Foll B. (2010). Noradrenergic alpha1 receptors as a novel target for the treatment of nicotine addiction. Neuropsychopharmacology.

[84] Erb S., Hitchcott P.K., Rajabi H., Mueller D., Shaham Y., Stewart J. (2000). Alpha-2 adrenergic receptor agonists block stress-induced reinstatement of cocaine seeking. Neuropsychopharmacology.

[85] Maldonado R., Valverde O., Berrendero F. (2006). Involvement of the endocannabinoid system in drug addiction. Trends Neurosci..

[86] Scherma M., Fadda P., Le Foll B., Forget B., Fratta W., Goldberg S.R., Tanda G. (2008). The endocannabinoid system: A new molecular target for the treatment of tobacco addiction. CNS Neurol. Disord. Drug Targets.

[87] Berrendero F., Robledo P., Trigo J.M., Martin-Garcia E., Maldonado R. (2010). Neurobiological mechanisms involved in nicotine dependence and reward: Participation of the endogenous opioid system. Neurosci. Biobehav. Rev..

[88] He Y., Galaj E., Bi G.H., Wang X.F., Gardner E., Xi Z.X. (2020). beta-Caryophyllene, a dietary terpenoid, inhibits nicotine taking and nicotine seeking in rodents. Br. J. Pharmacol..

[89] Xi Z.X., He Y., Shen H., Bi G.H., Zhang H.Y., Soler-Cedeno O., Alton H., Yang Y. (2023). GPR55 is expressed in glutamate neurons and functionally modulates nicotine taking and seeking in rats and mice. Res. Sq..

[90] De Vries T.J., de Vries W., Janssen M.C., Schoffelmeer A.N. (2005). Suppression of conditioned nicotine and sucrose seeking by the cannabinoid-1 receptor antagonist SR141716A. Behav. Brain Res..

[91] Le Foll B., Goldberg S.R. (2004). Rimonabant, a CB1 antagonist, blocks nicotine-conditioned place preferences. Neuroreport.

[92] Cohen C., Perrault G., Griebel G., Soubrie P. (2005). Nicotine-associated cues maintain nicotine-seeking behavior in rats several weeks after nicotine withdrawal: Reversal by the cannabinoid (CB1) receptor antagonist, rimonabant (SR141716). Neuropsychopharmacology.

[93] Le Foll B., Forget B., Aubin H.J., Goldberg S.R. (2008). Blocking cannabinoid CB1 receptors for the treatment of nicotine dependence: Insights from pre-clinical and clinical studies. Addict. Biol..

[94] Castane A., Berrendero F., Maldonado R. (2005). The role of the cannabinoid system in nicotine addiction. Pharmacol. Biochem. Behav..

[95] Cohen C., Perrault G., Voltz C., Steinberg R., Soubrie P. (2002). SR141716, a central cannabinoid (CB(1)) receptor antagonist, blocks the motivational and dopamine-releasing effects of nicotine in rats. Behav. Pharmacol..

[96] Butler K., Le Foll B. (2020). Novel therapeutic and drug development strategies for tobacco use disorder: Endocannabinoid modulation. Expert. Opin. Drug Discov..

[97] Carai M.A., Colombo G., Gessa G.L. (2005). Rimonabant: The first therapeutically relevant cannabinoid antagonist. Life Sci..

[98] Cohen C., Kodas E., Griebel G. (2005). CB1 receptor antagonists for the treatment of nicotine addiction. Pharmacol. Biochem. Behav..

[99] Simonnet A., Cador M., Caille S. (2013). Nicotine reinforcement is reduced by cannabinoid CB1 receptor blockade in the ventral tegmental area. Addict. Biol..

[100] Schindler C.W., Redhi G.H., Vemuri K., Makriyannis A., Le Foll B., Bergman J., Goldberg S.R., Justinova Z. (2016). Blockade of Nicotine and Cannabinoid Reinforcement and Relapse by a Cannabinoid CB1-Receptor Neutral Antagonist AM4113 and Inverse Agonist Rimonabant in Squirrel Monkeys. Neuropsychopharmacology.

[101] Liu J., Wu R., Li J.X. (2020). TAAR1 and Psychostimulant Addiction. Cell Mol. Neurobiol..

[102] Gainetdinov R.R., Hoener M.C., Berry M.D. (2018). Trace Amines and Their Receptors. Pharmacol. Rev..

[103] Zucchi R., Chiellini G., Scanlan T.S., Grandy D.K. (2006). Trace amine-associated receptors and their ligands. Br. J. Pharmacol..

[104] Borowsky B., Adham N., Jones K.A., Raddatz R., Artymyshyn R., Ogozalek K.L., Durkin M.M., Lakhlani P.P., Bonini J.A., Pathirana S. (2001). Trace amines: Identification of a family of mammalian G protein-coupled receptors. Proc. Natl. Acad. Sci. USA.

[105] Bunzow J.R., Sonders M.S., Arttamangkul S., Harrison L.M., Zhang G., Quigley D.I., Darland T., Suchland K.L., Pasumamula S., Kennedy J.L. (2001). Amphetamine, 3,4-methylenedioxymethamphetamine, lysergic acid diethylamide, and metabolites of the catecholamine neurotransmitters are agonists of a rat trace amine receptor. Mol. Pharmacol..

[106] Revel F.G., Moreau J.L., Gainetdinov R.R., Bradaia A., Sotnikova T.D., Mory R., Durkin S., Zbinden K.G., Norcross R., Meyer C.A. (2011). TAAR1 activation modulates monoaminergic neurotransmission, preventing hyperdopaminergic and hypoglutamatergic activity. Proc. Natl. Acad. Sci. USA.

[107] Bradaia A., Trube G., Stalder H., Norcross R.D., Ozmen L., Wettstein J.G., Pinard A., Buchy D., Gassmann M., Hoener M.C. (2009). The selective antagonist EPPTB reveals TAAR1-mediated regulatory mechanisms in dopaminergic neurons of the mesolimbic system. Proc. Natl. Acad. Sci. USA.

[108] Revel F.G., Moreau J.L., Gainetdinov R.R., Ferragud A., Velazquez-Sanchez C., Sotnikova T.D., Morairty S.R., Harmeier A., Groebke Zbinden K., Norcross R.D. (2012). Trace amine-associated receptor 1 partial agonism reveals novel paradigm for neuropsychiatric therapeutics. Biol. Psychiatry.

[109] Zhang Y., Li J.T., Wang H., Niu W.P., Zhang C.C., Zhang Y., Wang X.D., Si T.M., Su Y.A. (2021). Role of trace amine-associated receptor 1 in the medial prefrontal cortex in chronic social stress-induced cognitive deficits in mice. Pharmacol. Res..

[110] Liu J.F., Seaman R., Siemian J.N., Bhimani R., Johnson B., Zhang Y., Zhu Q., Hoener M.C., Park J., Dietz D.M. (2018). Role of trace amine-associated receptor 1 in nicotine’s behavioral and neurochemical effects. Neuropsychopharmacology.

[111] Sukhanov I., Dorofeikova M., Dolgorukova A., Dorotenko A., Gainetdinov R.R. (2018). Trace Amine-Associated Receptor 1 Modulates the Locomotor and Sensitization Effects of Nicotine. Front. Pharmacol..

[112] Wu R., Liu J., Johnson B., Huang Y., Zhang Y., Li J.X. (2022). Activation of trace amine-associated receptor 1 attenuates nicotine withdrawal-related effects. Addict. Biol..

[113] Matzeu A., Martin-Fardon R. (2021). Understanding the Role of Orexin Neuropeptides in Drug Addiction: Preclinical Studies and Translational Value. Front. Behav. Neurosci..

[114] al’Absi M., DeAngelis B., Nakajima M., Hatsukami D., Allen S. (2021). Early life adversity and appetite hormones: The effects of smoking status, nicotine withdrawal, and relapse on ghrelin and peptide YY during smoking cessation. Addict. Behav..

[115] Lemieux A.M., al’Absi M. (2018). Changes in circulating peptide YY and ghrelin are associated with early smoking relapse. Biol. Psychol..

[116] al’Absi M., Lemieux A., Hodges J.S., Allen S. (2019). Circulating orexin changes during withdrawal are associated with nicotine craving and risk for smoking relapse. Addict. Biol..

[117] Plaza-Zabala A., Maldonado R., Berrendero F. (2012). The hypocretin/orexin system: Implications for drug reward and relapse. Mol. Neurobiol..

[118] Bruijnzeel A.W. (2017). Neuropeptide systems and new treatments for nicotine addiction. Psychopharmacology.

[119] Kelly E.A., Fudge J.L. (2018). The neuroanatomic complexity of the CRF and DA systems and their interface: What we still don’t know. Neurosci. Biobehav. Rev..

[120] Grieder T.E., Herman M.A., Contet C., Tan L.A., Vargas-Perez H., Cohen A., Chwalek M., Maal-Bared G., Freiling J., Schlosburg J.E. (2014). VTA CRF neurons mediate the aversive effects of nicotine withdrawal and promote intake escalation. Nat. Neurosci..

[121] de Leon Reyes N.S., Sierra Diaz P., Nogueira R., Ruiz-Pino A., Nomura Y., de Solis C.A., Schulkin J., Asok A., Leroy F. (2023). Corticotropin-releasing hormone signaling from prefrontal cortex to lateral septum suppresses interaction with familiar mice. Cell.

[122] Hauger R.L., Grigoriadis D.E., Dallman M.F., Plotsky P.M., Vale W.W., Dautzenberg F.M. (2003). International Union of Pharmacology. XXXVI. Current status of the nomenclature for receptors for corticotropin-releasing factor and their ligands. Pharmacol. Rev..

[123] Koob G.F. (2010). The role of CRF and CRF-related peptides in the dark side of addiction. Brain Res..

[124] Koob G.F., Buck C.L., Cohen A., Edwards S., Park P.E., Schlosburg J.E., Schmeichel B., Vendruscolo L.F., Wade C.L., Whitfield T.W. (2014). Addiction as a stress surfeit disorder. Neuropharmacology.

[125] George O., Ghozland S., Azar M.R., Cottone P., Zorrilla E.P., Parsons L.H., O’Dell L.E., Richardson H.N., Koob G.F. (2007). CRF-CRF1 system activation mediates withdrawal-induced increases in nicotine self-administration in nicotine-dependent rats. Proc. Natl. Acad. Sci. USA.

[126] Cohen A., Treweek J., Edwards S., Leao R.M., Schulteis G., Koob G.F., George O. (2015). Extended access to nicotine leads to a CRF1 receptor dependent increase in anxiety-like behavior and hyperalgesia in rats. Addict. Biol..

[127] Bruijnzeel A.W., Prado M., Isaac S. (2009). Corticotropin-releasing factor-1 receptor activation mediates nicotine withdrawal-induced deficit in brain reward function and stress-induced relapse. Biol. Psychiatry.

[128] Nestler E.J., Carlezon W.A. (2006). The mesolimbic dopamine reward circuit in depression. Biol. Psychiatry.

[129] Wee S., Koob G.F. (2010). The role of the dynorphin-kappa opioid system in the reinforcing effects of drugs of abuse. Psychopharmacology.

[130] Albores-Saavedra J., Gorraez de la Mora T., de la Torre-Rendon F., Gould E. (1990). Mixed medullary-papillary carcinoma of the thyroid: A previously unrecognized variant of thyroid carcinoma. Hum. Pathol..

[131] Al-Hasani R., McCall J.G., Bruchas M.R. (2013). Exposure to chronic mild stress prevents kappa opioid-mediated reinstatement of cocaine and nicotine place preference. Front. Pharmacol..

[132] Muschamp J.W., Hollander J.A., Thompson J.L., Voren G., Hassinger L.C., Onvani S., Kamenecka T.M., Borgland S.L., Kenny P.J., Carlezon W.A. (2014). Hypocretin (orexin) facilitates reward by attenuating the antireward effects of its cotransmitter dynorphin in ventral tegmental area. Proc. Natl. Acad. Sci. USA.

[133] Ito H., Fukatsu N., Rahaman S.M., Mukai Y., Izawa S., Ono D., Kilduff T.S., Yamanaka A. (2023). Deficiency of orexin signaling during sleep is involved in abnormal REM sleep architecture in narcolepsy. Proc. Natl. Acad. Sci. USA.

[134] Beckenstrom A.C., Coloma P.M., Dawson G.R., Finlayson A.K., Malik A., Post A., Steiner M.A., Potenza M.N. (2023). Use of experimental medicine approaches for the development of novel psychiatric treatments based on orexin receptor modulation. Neurosci. Biobehav. Rev..

[135] Yaeger J.D.W., Krupp K.T., Jacobs B.M., Onserio B.O., Meyerink B.L., Cain J.T., Ronan P.J., Renner K.J., DiLeone R.J., Summers C.H. (2022). Orexin 1 Receptor Antagonism in the Basolateral Amygdala Shifts the Balance From Pro- to Antistress Signaling and Behavior. Biol. Psychiatry.

[136] Hollander J.A., Lu Q., Cameron M.D., Kamenecka T.M., Kenny P.J. (2008). Insular hypocretin transmission regulates nicotine reward. Proc. Natl. Acad. Sci. USA.

[137] Lengel D., Kenny P.J. (2023). New medications development for smoking cessation. Addict. Neurosci..

[138] Mellentin A.I., Finn S.W., Skot L., Thaysen-Petersen D., Mistarz N., Fink-Jensen A., Nielsen D.G. (2023). The effectiveness of oxytocin for treating substance use disorders:A systematic review of randomized placebo-controlled trials. Neurosci. Biobehav. Rev..

[139] Van Hedger K., Bershad A.K., Lee R., de Wit H. (2020). Effects of Intranasal Oxytocin on Stress-Induced Cigarette Craving in Daily Smokers. Nicotine Tob. Res..

[140] McReynolds J.R., Wolf C.P., Starck D.M., Mathy J.C., Schaps R., Krause L.A., Hillard C.J., Mantsch J.R. (2023). Role of mesolimbic cannabinoid receptor 1 in stress-driven increases in cocaine self-administration in male rats. Neuropsychopharmacology.

[141] Panin F., Lintas A., Diana M. (2014). Nicotine-induced increase of dopaminergic mesoaccumbal neuron activity is prevented by acute restraint stress. In vivo electrophysiology in rats. Eur. Neuropsychopharmacol..

[142] Enrico P., Sirca D., Mereu M., Peana A.T., Mercante B., Diana M. (2013). Acute restraint stress prevents nicotine-induced mesolimbic dopaminergic activation via a corticosterone-mediated mechanism: A microdialysis study in the rat. Drug Alcohol Depend..

[143] Bilkei-Gorzo A., Racz I., Michel K., Darvas M., Maldonado R., Zimmer A. (2008). A common genetic predisposition to stress sensitivity and stress-induced nicotine craving. Biol. Psychiatry.

[144] Cadet J.L. (2016). Epigenetics of Stress, Addiction, and Resilience: Therapeutic Implications. Mol. Neurobiol..

[145] al’Absi M., Nakajima M., Lemieux A. (2018). Impact of early life adversity on the stress biobehavioral response during nicotine withdrawal. Psychoneuroendocrinology.

[146] Lee A.M., Calarco C.A., McKee S.A., Mineur Y.S., Picciotto M.R. (2020). Variability in nicotine conditioned place preference and stress-induced reinstatement in mice: Effects of sex, initial chamber preference, and guanfacine. Genes Brain Behav..

[147] al’Absi M., Nakajima M., Allen S., Lemieux A., Hatsukami D. (2015). Sex differences in hormonal responses to stress and smoking relapse: A prospective examination. Nicotine Tob. Res..

[148] Yararbas G., Keser A., Kanit L., Pogun S. (2010). Nicotine-induced conditioned place preference in rats: Sex differences and the role of mGluR5 receptors. Neuropharmacology.

[149] Xu J., Azizian A., Monterosso J., Domier C.P., Brody A.L., Fong T.W., London E.D. (2008). Gender effects on mood and cigarette craving during early abstinence and resumption of smoking. Nicotine Tob. Res..

[150] Bohadana A., Nilsson F., Rasmussen T., Martinet Y. (2003). Gender differences in quit rates following smoking cessation with combination nicotine therapy: Influence of baseline smoking behavior. Nicotine Tob. Res..

[151] Ward K.D., Klesges R.C., Zbikowski S.M., Bliss R.E., Garvey A.J. (1997). Gender differences in the outcome of an unaided smoking cessation attempt. Addict. Behav..

[152] Wetter D.W., Kenford S.L., Smith S.S., Fiore M.C., Jorenby D.E., Baker T.B. (1999). Gender differences in smoking cessation. J. Consult. Clin. Psychol..

[153] McKee S.A., Maciejewski P.K., Falba T., Mazure C.M. (2003). Sex differences in the effects of stressful life events on changes in smoking status. Addiction.

[154] Li S., Zou S., Coen K., Funk D., Shram M.J., Le A.D. (2014). Sex differences in yohimbine-induced increases in the reinforcing efficacy of nicotine in adolescent rats. Addict. Biol..

[155] al’Absi M. (2006). Hypothalamic-pituitary-adrenocortical responses to psychological stress and risk for smoking relapse. Int. J. Psychophysiol..

[156] al’Absi M., Nakajima M., Grabowski J. (2013). Stress response dysregulation and stress-induced analgesia in nicotine dependent men and women. Biol. Psychol..

[157] Wardle M.C., Munafo M.R., de Wit H. (2011). Effect of social stress during acute nicotine abstinence. Psychopharmacology.

[158] Schepis T.S., Tapscott B.E., Krishnan-Sarin S. (2016). Stress-related increases in risk taking and attentional failures predict earlier relapse to smoking in young adults: A pilot investigation. Exp. Clin. Psychopharmacol..

[159] Lee S.E., Kim C.W., Im H.B., Jang M. (2021). Patterns and predictors of smoking relapse among inpatient smoking intervention participants: A 1-year follow-up study in Korea. Epidemiol. Health.

[160] Megias-Robles A., Perea-Baena J.M., Fernandez-Berrocal P. (2020). The protective role of emotional intelligence in smoking relapse during a 12-month follow-up smoking cessation intervention. PLoS ONE.

[161] Koob G.F., Caine S.B., Parsons L., Markou A., Weiss F. (1997). Opponent process model and psychostimulant addiction. Pharmacol. Biochem. Behav..

[162] Epping-Jordan M.P., Watkins S.S., Koob G.F., Markou A. (1998). Dramatic decreases in brain reward function during nicotine withdrawal. Nature.

[163] Markou A., Kosten T.R., Koob G.F. (1998). Neurobiological similarities in depression and drug dependence: A self-medication hypothesis. Neuropsychopharmacology.

[164] Ashare R.L., Lerman C., Cao W., Falcone M., Bernardo L., Ruparel K., Hopson R., Gur R., Pruessner J.C., Loughead J. (2016). Nicotine withdrawal alters neural responses to psychosocial stress. Psychopharmacology.

[165] Hogle J.M., Kaye J.T., Curtin J.J. (2010). Nicotine withdrawal increases threat-induced anxiety but not fear: Neuroadaptation in human addiction. Biol. Psychiatry.

[166] al’Absi M., Hatsukami D., Davis G.L. (2005). Attenuated adrenocorticotropic responses to psychological stress are associated with early smoking relapse. Psychopharmacology.

[167] al’Absi M. (2018). Stress and Addiction: When a Robust Stress Response Indicates Resiliency. Psychosom. Med..

[168] Shaw D., al’Absi M. (2008). Attenuated beta endorphin response to acute stress is associated with smoking relapse. Pharmacol. Biochem. Behav..

